# Epidemiology of musculoskeletal injuries in a population of harness Standardbred racehorses in training

**DOI:** 10.1186/1746-6148-10-11

**Published:** 2014-01-10

**Authors:** Andrea Bertuglia, Michela Bullone, Federica Rossotto, Mauro Gasparini

**Affiliations:** 1 Department of Veterinary Science, Università di Torino, via Leonardo da Vinci 44, 10095 Grugliasco, (TO), Italy; 2 Department of Clinical Sciences, Université de Montréal, 3200 rue Sicotte, Saint-Hyacinthe J2S 2 M2 Quebec, Canada; 3 Department of Mathematical Sciences, Politecnico di Torino, Corso Duca degli Abruzzi 24, 10129 Torino, Italy

**Keywords:** Standardbred racehorses, Epidemiology, Trotter, Musculoskeletal injuries, Exercise-related injuries

## Abstract

**Background:**

There is a substantial paucity of studies concerning musculoskeletal injuries in harness Standardbred racehorses. Specifically, little is known about the epidemiology of exercise-related musculoskeletal injuries. Most studies on this subject involve Thoroughbred racehorses, whose biomechanics and racing speed differ from Standardbred, making comparisons difficult. Here, a population of Standardbred racehorses trained at the same racecourse was studied over four years and a classification system for exercise-related musculoskeletal injuries was designed. The incidence rates of musculoskeletal injuries causing horses’ withdrawal from training for 15 days or longer were investigated. A mixed-effects Poisson regression model was used to estimate musculoskeletal injury rates and to describe significance of selected risk factors for exercise-related injuries in this population.

**Results:**

A total of 356 trotter racehorses from 10 different stables contributed 8961 months at risk of musculoskeletal injuries. Four-hundred-and-twenty-nine injuries were reported and classified into 16 categories, based on their aetiology and anatomical localisation. The overall exercise-related injury rate was 4.79 per 100 horse months. When considering risk factors one by one in separate univariable analyses, we obtained the following results: rates did not differ significantly between genders and classes of age, whereas one driver seemed to cause fewer injuries than the others. Racing speed and racing intensity, as well as recent medical history, seemed to be significant risk factors (p < 0.001), while being shod or unshod during racing was not. On the other hand, when pooling several risk factors in a multivariable approach, only racing intensity turned out to be significant (p < 0.001), since racing speed and the racing intensity were partially confounded, being strongly correlated to one another.

**Conclusion:**

Characterizing epidemiology of exercise-related musculoskeletal injuries in trotter racehorses provides baseline incidence rate values. Incidence rates of stress fracture are lower in Standardbreds compared to Thoroughbreds, whereas the opposite is true for tendon and suspensory ligament injuries. In addition to identification of risk factors for musculoskeletal injuries among Standardbred racehorses, results suggest that racing intensity seems to be a protective predictor of risk and recent medical history could be used to identify horses at risk of injury.

## Background

Harness racing is a popular racehorse discipline, developed from a traditional recreational activity, in which horses race at a specific gait (trot) and they pull a two-wheeled cart called *sulky*. Musculoskeletal injuries (MSI) are the primary cause of reduced training days and racehorse wastage in Standardbred racehorses (STBR) as well as in Thoroughbred racehorses (TBR) [1-6]. But, only a few research studies have attentively focused on STBR specific problems [7-10], such as middle carpal joint injuries [8] and proximal sesamoid bone fractures [9]. Large-scale studies on exercise-related MSI in this population are missing, compared to TBR, probably because in STBR catastrophic events are rare during competitions, thus not raising welfare concerns on racecourse safety. Despite the differences in their running style, data from studies on TBR are commonly transferred to STBR, based on the assumption of a theoretical similar exposure to common risk factors. However, different loading patterns may cause dissimilar injury profiles in horses employed for different disciplines [10,11], much as in human athletes practicing different sports [12].

In TBR, age and gender have been shown to be specific risk factors for injury development, with older animals being at increased risk of tendon and ligament traumas [13-17] and females being more at risk of stress fractures [3,5]. Racetrack surface [2,3], age [1,3,6], gender [1,6,16,17], season [3,5,16], trainer [4,5,15-17], pre-existing pathologies [18-20], accumulation of exercise at racing speed [21,22], average distance run per week [23,24], laying-up periods [2,22] and inter-race interval [2,22] have all been studied in TBR populations, with contradictory results. Also, shoeing techniques received great attention [19,25,26]; particularly one of them, the toe grab, is a recognized risk factor for MSI in TBR due to the alteration of the horse’s gait at high speed [19,26]. It is generally accepted that variations exist between different racetracks [3], other than different types of race in TBR [5,27,28].

Few data exist in the literature about the effect of these factors on STBR and their investigation would be worthwhile. In STBR training, the driver has been reported to be a risk factor for selected MSI in young animals [8]. STBR are rarely exposed to different environments because similar surfaces are commonly found in all racecourses and race distances are similar all over the world. However, a number of specific risk factors need to be considered, like racetrack banking at the racecourse curves [7]. Methods employed in STBR training are all conceptually similar [8,10], although the volume and the intensity of training for young animals are variable and they could be considered risk factors for MSI. Generally STBR perform more frequently than TBR during their athletic careers. The average annual racing intensity could represent an indirect measure of the intensity of exercise since a higher number of races is considered a potential risk factor for MSI. Preliminary existing data reported that STBR winning from 1 to 7 races in one season suffer twice as many MSI than others [10]. In STBR practice it is commonly observed that some horses are presented for racing barefoot at the level of forelimbs or both at the front and rear. The strategy of racing without shoes in order to improve the performance was pinpointed as a risk factor for MSI [29].

In many horses, abnormalities in trotting gait are commonly observed concurrently to the increase of speed during training. These gait abnormalities include several interferences between forelimbs and ipsilateral hindlimbs and breaks into a gallop. These issues are commonly addressed with the use of corrective harnesses intervening on gait or, when they are allocated to lameness, it takes veterinary opinion. Although veterinarians are often called upon to solve gait problems through the use of local treatments or joint medications, there are no studies defining the relationship between recent treatments and MSI in STBR.

Epidemiological studies in STBR would benefit from an objective and specific categorization of exercise-related injuries. Indeed, studies focusing on single lameness events and days of training suspension could be misleading given the fact that multiple lameness episodes may result from a single pathology. For this reason, large-scale studies based on opinion surveys and retrospective interviews with non-veterinary staff [10] need to be attentively analysed in order to avoid data misinterpretation. Studies focusing on lameness cases in STBR are better defined by veterinary reports because trainer opinions are potentially misleading, since many horses are normally trained with gait problems, and those imperfections are related to a sign of immaturity in the trotting efficiency rather than orthopaedic pain. As stated in recent research [6], classification systems of exercise-related injuries should describe diseases on the basis of common pathogenesis, repetition and severity of injury, rather than on simple anatomical considerations. These features would make a classification suitable for studies aimed at identifying risk factors for specific diseases.

In the present study, we outline the occurrence of MSI in a population of harness STBR in training at the racetrack of Turin (Italy) over a four-year period, with the aim of estimating the specific incidence rate (IR) of MSI and defining the importance of some possible risk factors.

## Methods

This study was approved by the Ethical Committee of the University of Turin.

### Animal selection

A retrospective open-cohort observational study was conducted. Data regarding horse management, daily training regimen and exercise-related injuries were collected for harness STBR in training at the racecourse of Turin-Vinovo from the 1st January 2008 to the 31st December 2011.

A sample of 10 professional drivers was studied, based on data availability. All the horses trained by these drivers throughout the study period were enrolled. Some animals were retired because of age, economic considerations, exercise-related injuries or severe medical conditions. The animals had to meet the following inclusion criteria: 1) having been trained for a minimum of 6 months, 2) availability of comprehensive veterinary records for all the considered time, 3) belonging to a stable where first-opinion veterinary care was provided on a regular basis and exclusively by the first author working team, and 4) having spent the time at risk at the selected racetrack under the direction of the same driver continuously.

### Data collection

The data on orthopaedic pathologies and training for each horse were obtained both by consulting veterinary records and by interviewing the drivers. Signalment data were obtained from their passports and their racing career details from the ANACT (*Associazione Nazionale Allevatori Cavallo Trottatore*) online database [30]. For each subject, the following data were recorded: date of birth based on an arbitrary date adjusted to 1st January of the year of birth, gender, beginning and duration of the observational period, the driver of the animal, the maximum racing speed obtained in the last one thousand metres of races and the average annual racing intensity.

The data collection for each animal started on the day the horse entered the stable to be trained and continued until the study ended or until the horse was retired from racing, died or changed driver. The injuries were recorded and classified into categories. The dates of injuries, affected leg(s) and shoeing technique during races were registered. All clinical examinations for lameness, diagnostic imaging report, and medical treatments adopted by veterinarians to manage gait problems were recorded in an individual report. The days of effective training were considered to derive the time at risk and to define the IR of MSI. Months were employed as time measuring units. Regarding rest periods, months were rounded to the nearest integer, zero included.

### Case definition and injury classification system

MSI were defined as exercise-related lameness episodes occurring as a consequence of overloading or cumulative stress. MSI occurring both during training and racing were included in the study. Self-inflicted lacerations and direct trauma due to interferences occurring during fast exercise were counted, whereas others injuries caused by accidental trauma (e.g. major collision with a fence or an object) were not. MSI had to be confirmed by diagnostic imaging (radiology, ultrasonography or scintigraphy) and specific diagnostic analgesia, when possible. MSI were considered only if they interfered with the planned training program (we considered a training interference the complete suspension of exercise) for at least 15 days and when both clinical diagnosis and proper anatomical localization were identified. Multiple injuries affecting the same horse during the study period were included in the analysis. Re-occurrence of the same lesion in the same anatomical structure was classed as re-injury and considered as a separate event when calculating IR of MSI, while bilateral injuries occurring simultaneously accounted for one case.

Injuries were classified in categories to facilitate the dataset construction. Each category of MSI was considered only when the related number of injuries accounting for it was greater than 1% of the total number of MSI in the studied population. Sixteen categories of MSI were defined, on the basis of anatomical localization, repeatability and pathophysiology (see Additional file 1).

### Risk factors

The following potential risk factors for developing MSI were considered: AGE, GENDER, DRIVER, RACING SPEED, RACING INTENSITY, RACING SHOD, and MEDICAL TREATMENTS.

Two classes of the variable AGE were defined: young (horses 18-month to 4-year old) and adult (horses 5 to 10-year old). Animals over 10 years are compulsorily retired from racing, whereas animals under 18 months are not generally trained. The age of 4 years was chosen as cut-off value as it is commonly the age at which STBR reach the maximum racing speed.

Males, females and geldings were studied as variable GENDER. Castrated horses were defined as geldings for the full duration of the study, since the majority of males are castrated in the first year of training.

We combined into the variable DRIVER the complexity of training methodologies and related issues that any individual driver decides to adopt. Drivers also influence parameters difficult to quantify, such as the capability of identifying subtle lameness episodes and their subsequent decisions to suspend training or to recur to corrective harness to improve horse gait.

RACING SPEED of the horse was determined based on its personal best time and it was defined into five different categories, considering the animals performing faster (<72 sec/km) as top STBR, those performing ≥72 sec/km and <74 sec/km as elite STBR, those racing ≥74 sec/km and <76 sec/km as medium level STBR, those performing ≥76 sec/km and <78 sec/km as low level STBR and the group of STBR performing ≥78 sec/km as not qualified STBR. The time of 78 sec/km is considered in Italy as the minimum qualification time to participate in a race program.

RACING INTENSITY was calculated as the mean number of races a horse participated in during a year. Four classes of race numbers were determined, based on an average number of one race a month. Class 1 was defined as 0 to 4 races per year, class 2 was defined as 5 to 11 races per year, class 3 as 12 to 22 races per year and class 4 as 23 to 32 races per year.

MEDICAL TREATMENTS referred to the veterinary treatments that STBR received, including the following treatments at the same level of importance: joint medications, regional medication and mesotherapy (multiple injections of pharmaceutical medications into subcutaneous space) for back pain and systemic anti-inflammatory medication. All veterinary treatments were considered according to the doping rules in Italy, because horses receiving such treatments in an inappropriate way are excluded from the race program. Treatments were considered only in the 30 days before a MSI occurred in our population, to establish a strong correlation between the two events.

The variable RACING SHOD refers to whether horses race with some plastic or aluminium shoes rather than without shoes in competitions, either on forelimbs only or both forelimbs and hindlimbs. The variables RACING SHOD and MEDICAL TREATMENTS are restricted variables for which only the qualified horses and injured horses, respectively, were categorised.

### Data processing and Statistical Analysis

All data on individual horses and specific injuries were entered into a customized database. The data were then exported to the statistical software R (R version 2.15.3, R development Core Team, 2011), summarized in appropriate multidimensional contingency tables and analysed using Poisson regression tools. In particular, artificial statistical units were made up for Poisson regression as described next. Given a certain combination of factor levels (e.g, GENDER = female / RACING SPEED = low level / RACING INTENSITY = 0 to 4 races), the corresponding horses were identified and merged together in one such artificial unit. The artificial unit was then assigned a number of injuries computed as the overall number of injuries of the corresponding horses and training time computed as the sum of the training times of the corresponding horses.

**Figure 1:**
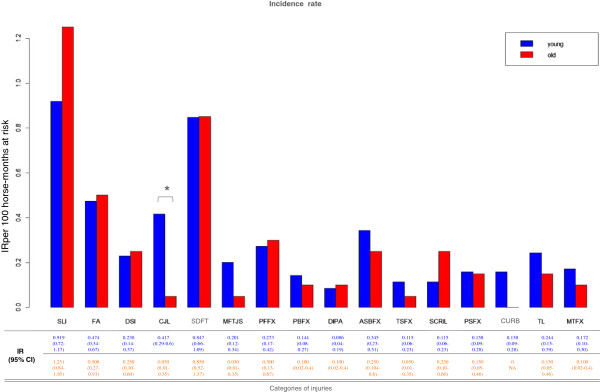
**Estimated IRs for injuries by age groups.** Histogram of estimated IR for categories of injuries in a cohort of harness STBR, by categories of injuries in the groups of young (<4 years old) and adult (>4 years old) horses.

Univariable Poisson regression analyses, only focused on risk factor AGE, were initially conducted for each category of MSI (Additional file 2: Table S1) and summarized in Figure 1. Overall IRs related to categories of MSI were directly calculated and exact 95% CIs were obtained following the Ulm method [31].

Univariable Poisson regression models on the whole population of horses were implemented for each risk factor, with the aim to screen significant ones. IRs for each level of each factor, IRRs (incidence rates ratio) and associated 95% CIs were assessed and are shown in Additional file 3. Analysis of all types of injuries pooled together, limited to particular subsets of the population, was conducted with the same method. The first subset of horses refers to those who ran at least one race, so that variable RACING SHOD could be determined, whereas the second subset are those suffering at least one injury during the observational period, so that the variable MEDICAL TREATMENTS could be analysed.

Risk factors that reached an acceptable univariable significance threshold (p < 0.05) were retained in a more stringent multivariate Poisson mixed regression model (Additional file 4) and tested again for significance. In such multivariable analysis, the variable DRIVER was introduced as a frailty random effect, to provide more accurate estimates of the other retained predictors, which were considered as fixed effects. Our interest in variable DRIVER was two-fold. On one hand, we wanted to check whether there were significant risk differences between those ten specific drivers who happened to run our horses; on the other hand, we realized that these ten drivers could also be seen as random effects coming from an hyperpopulation of drivers. Driver was treated as a fixed effect in the univariable analysis and as a random effect in the multivariable one.

## Results

A total of 356 harness STBR in training were selected on the basis of the inclusion criteria and contributed 8961 months at risk for MSI. A total of 331 (92.98%) horses sustained 429 injuries. While 397 (92.54%) injuries were classified as individual events, 32 (7.46%) were considered re-injuries. In our racehorse population, 234 (65.73%) STBR had one injury, while 97 (27.25%) had more than one. The overall IR in our population was 4.79 injuries per 100 horse months.

In Additional file 1 the categories of exercise-related MSI are defined on the basis of the clinical findings. In Figure 1 overall IRs by different categories of injuries and univariable analysis results for age classes are shown. When looking at the most representative category, the SLI was the most frequently reported, followed by SDFT with an IR of 0.99 and 0.84 per 100 horse months, respectively. Despite the high IR variability between young and adult horses shown in Additional file 3, only results for CJL were significant (p = 0.037).

IRs within groups determined by the levels of our risk factors of interest are shown in Additional file 3. In addition, Additional file 3 contains IRR, which are measures of differential risk with respect to a reference group (in our case, an arbitrary level of the risk factor).

Results of the univariable screening of risk factors for MSI can be summarized as follows:

a) The MSI IRs in our population do not vary significantly with AGE, whereas geldings are at reduced risk with respect to males (IRR = 0.71, CI = 0.54-0.94, p = 0.018).

b) There is some significant DRIVER effect, i.e. driver 3 shows the lowest IR and a better ability to modulate exercise intensity (IRR = 0.57, CI = 0.34-0.97, p = 0.039). In the group of 10 drivers considered there is a high variability in the number of wins and earning, but this difference does not reflect a higher incidence of MSI to the drivers who have greater success (Additional file 5: Table S2).

c) A high value of IR of MSI was observed for the lowest level of variable *RACING SPEED*, i.e. “not-qualified STBR”. The IR then decreases progressively from this lowest level (IR = 10.29) to the upper classes of “elite-STBR” (IR = 3.65) and “top-STBR” (IR = 3.89). Tests of significance of the three highest levels of RACING SPEED confirm strong significance of these comparisons (p < 0.001).

d) The results regarding the RACING INTENSITY variable show that the IRs of MSI decrease as the number of races increases. Tests of significance of the three highest levels of annual racing intensity reached significant results (adjusted p < 0.001) with respect to the reference level (“0-4 races per year”).

e) The variable RACING SHOD pertains only to the subset of the population made up of horses that successfully completed the qualification race and had run more than one race. Similarly, variable MEDICAL TREATMENTS only included horses that suffered at least one MSI. In the first case, no significant values were observed, whereas, in the latter case, the analysis reveals strongly significant differences among the two levels of the variable MEDICAL TREATMENTS (p < 0.001), as shown in Additional file 3.

Based on univariable screening we then selected, only among the variables studied over the whole population, the variables with a significant univariable effect on the response variable (p < 0.05). These variables were retained in a multivariable mixed Poisson regression model (Additional file 4), in which variable DRIVER has been considered as a random effect, as described previously.

In our analysis, the DRIVER and, indirectly, the training strategy adopted were not significantly associated with the occurrence of MSI in STBR in the multivariate model.

In this model, variables GENDER and RACING SPEED turned out to be no longer significant; similarly, the estimate of the variance of the random effect DRIVER is null, making the influence of the driver effect essentially disappear.

On the other hand, the variable RACING INTENSITY maintained significance also in this multivariable, more stringent, approach (p < 0.001), with the “23 to 32 races per year” level showing significantly smaller IRR than lower intensities.

A possible explanation for these results is that RACING INTENSITY acts as a representation of the “healthy horse” effect, making correlated variables such as RACING SPEED not significant when introduced in the same explanatory pool of predictors. These results are confirmed by the significance of post-hoc tests of dependence of RACING INTENSITY on RACING SPEED. If we retain as response the original number of races per year, the p-values of the ANOVA test on RACING SPEED as well as the Kruskal-Wallis test are approximately zero.

## Discussion

This is the first study aimed at estimating IRs of major MSI in a population of harness STBR in training and at evaluating the significance of some horse and training-related risk factors for MSI, using a longitudinal retrospective cohort study conducted over a 4-year observational period.

### Prevalence of musculoskeletal injuries

Large-scale epidemiological studies are receiving great attention thanks to their potential to identify causal associations between exercise, orthopaedic injury incidence and related risk factors in racehorse populations [32]. Nevertheless, the ability of an optimum training regimen in preventing injuries remains elusive [2,32].

The overall IR for MSI in harness STBR are barely comparable with IR reported in the literature on TBR. However, the prevalence of specific injuries in STBR shows consistent differences with those reported in TBR during training. As in human sports, the anatomical distribution of overuse injuries was influenced by the loading patterns of the specific discipline [12]. In a population of National Hunt TBR, overall fractures had an IR of 1.1/100 horse months, with an IR of 1.7/100 horse months for SDFT and 0.23/100 horse months for SLI [16]. According to a retrospective study conducted in the UK flat racing, the most represented categories of injuries in TBR are pelvic and tibial stress fractures followed by lateral condylar fractures of the third metacarpal and metatarsal bone [5]. While stress fractures and SDFT are the commonest exercise-related lesions at all in TBR [2,3,17,33], SLI was the most common injury observed in our study (IR = 0.99/100 horse months), followed by SDFT (IR = 0.84/100 horse months). Damage to these structures accounted for 38.3% of the overall MSI in our study. Tibial stress fractures are uncommon injuries in STBR, while lateral condylar fractures and biaxial proximal sesamoid bone fractures were not observed.

Other than racecourse designs, racetrack surfaces, maximum racing speed and gait differences are likely to account for such differences between the two breeds. The biomechanics of “flying trot” and the slower speed compared to high-speed gallop seems to reduce the likelihood of bone failure accidents. The limited vertical oscillation of the centre of mass, during trot, generates a reduced impact in front legs during the stance phase of the stride [34]. Secondarily, the storage of elastic energy into the collagenous tissues along the spine, gluteal and hamstring muscles at gallop, could potentially explain the high incidence of pelvic and tibia fractures sustained in TBR [35].

In harness STBR, the improvement of gait mechanics and efficiency leading finally to “flying trot” is normally acquired with age and training [36]. This gait optimization is likely to increase the load on the suspensory apparatus, even if it does not lead to an increased likelihood of damages proportionally to the speed of the animal. The absence of catastrophic injuries like suspensory breakdown in harness racing could be related to the slower speed, to the position of the centre of mass, more caudal compared to TBR, and to the characteristic of the racecourse bank on the curves [7,37]. At higher speed, differences in limb inclination related to a slight bank on the curves, generate more centripetal forces at the ground, increasing limb rotational movements, which may increase the risk of injuries [37]. An increase in the racetrack bank on the curves was protective for MSI in an observational study on STBR racecourses [7]. Finally, the high prevalence of overuse injuries of the suspensory ligament could stem from a high rate of compensatory lameness in STBR, due to an improper load distribution in this symmetrical gait [38].

A percentage of 92.9% horses starting training in our study sustained at least one musculoskeletal injury during their observation time. A single study on TBR defined that approximately 25% of horses in training sustain a significant MSI per calendar year [3]. The data from the two specialties are not easily comparable in this form. Moreover, the exclusion from our study of cases of lameness not related to a consistent period of rest, contributed to an underestimation of the number of injuries.

### Risk factors

Although many variables have shown to be important in exercise-related MSI rates in TBR, only few of them are realistically applicable to harness racing because of the differences in both training and management between these populations. In our study we took into account arbitrary variables derived from the experience of veterinarians and professional drivers working with STBR. When injuries were studied together and separately, AGE did not represent a risk factor for MSI in our population. The different methods of training adopted by drivers are considered in the literature [10,21] to be an essential factor influencing racehorse injury rates. We have mixed results about this: on one hand, some significant differences between drivers are observed when the drivers are compared one against the other in a univariable analysis. On the other hand, the variability of the drivers becomes insignificant when inserted in a multivariable analysis in the presence of other predictors.

Specific training methods and cumulative distance of fast training were not evaluated as risk factors for MSI *per se* in our study. Supporting our findings, a study on middle carpal joint lameness in STBR showed that a higher incidence of third carpal bone pathology was not related to more intense exercise programs [8]. In contrast, epidemiological studies in young TBR demonstrated that different trainers were correlated with different MSI IRs [5,6]. Faster performing STBR showed a progressive significant reduction of MSI rates in our study. Moreover, intense training techniques, commonly employed for athletes performing faster, did not result in an increased likelihood of exercise-related injuries. This is potentially due to the “healthy horse effect” [2], leading to the prompt exclusion of those animals experiencing an elevated number of injuries from the racing population. MSI thus represent a consistent limit for equine athletes to reach their maximum performance level. Successful initial exercise adaptability is a key step in an elite racehorse’s career and our work seems to suggest that it has more influence than variables related to training intensity and racing intensity on exercise-related injury occurrence. Our findings seem to confirm that the more exercise horses accumulate, the less likely they are to sustain injuries [18,39]. This relationship had been shown previously, with high intensity exercise seemingly being protective for stress fracture development [40,41]. Genetic differences could be advocated, possibly influencing susceptibility to injuries in certain bloodlines of STBR, as postulated in men [42]. In our study, the RACING SPEED represented a significant risk factor for MSI occurrence only in the univariable analysis but not in the multivariable analysis. Variables RACING SPEED and RACING INTENSITY are naturally strongly correlated. It is therefore not surprising that, in the multivariable analysis, only one of the two variables, RACING INTENSITY, remains significant. However a bias could have been introduced as racing programs for high-level athletes are better modulated.

Data regarding catastrophic injury occurrence in TBR focused on pre-existing pathologies as risk factors for MSI development [18]. In a study on SDFT, horses receiving veterinary examinations for a tendon problem had a 10-fold increased risk compared to others to sustain tendon injuries in the following 60 days [14]. Results from the current study suggest that veterinary treatments to solve gait problems and subtle lameness increase the risk of major MSI in STBR. Animals receiving medical treatments had a 2.6-fold increased risk compared to others to sustain a MSI in the following 30 days, not necessarily with the same location of the treatment*.* This is an important finding, as it is a risk factor that is potentially modifiable*.* The beneficial effect of veterinary treatment to resolve lameness needs to be evaluated in the light of this increased risk, because we could speculate that these horses most likely already had some form of injury*.*

The tendency for STBR to race unshod is because it is thought to allow the animal to perform faster. However, the unshod foot cannot neutralize the impact with the ground during fast trot [43]. In our study, horses racing unshod did not show a different risk of major MSI occurrence compared to horses racing with shoes.

### Methodological considerations and limitations

Our study may not be representative of the general STBR population, as the horses studied were all from the same racetrack where few blood lineages are overrepresented compared to others. The prolonged study period represents a strength of our work. Despite some variation in the frequency with which veterinary examinations were performed, a frequency of twice a week was kept through all the study period, increasing the likelihood of detection of even subtle lameness. However, horses with mild lameness may not have been referred to receive veterinary attention and mild injuries may have been unreported. The trainer selection was based on the quality of authorized veterinary interventions, which represented a way to mitigate bias. Some bias was introduced by the selection of MSI and animals. An arbitrary evidence-based classification system of MSI was adopted, intended for use in our observational studies. We decided to focus the study on major exercise-related MSI, associated to a consistent period of rest. Indeed, some common orthopaedic conditions requiring only a few days of rest like sole bruising, recurrent myopathies and splint bone pain were considered as non-cases. Also, uncommon conditions such as carpal canal haemorrhage were excluded due to their low incidence in our population. Moreover, not all the horses could be submitted to a complete diagnostic process in order to obtain a precise definition of the problem itself, principally for economic reasons. This consideration caused minor case loss in our data. The decision to restrict observations to STBR with at least 6 months of training, required necessary to define a cumulative stress, could introduce a bias on rate measurement. The very low number of horses belonging to this group and the fair distribution of those animals between the drivers mitigate this potential selection bias.

## Conclusions

This study provides accurate estimates of exercise-related MSI IR associated with training in a population of harness STBR trained in a selected racecourse. Considerations about these specific findings need to be taken into account when attempting to extrapolate what is reported here to the wider population. We have shown that incidence rates of MSI in STBR differ consistently from those observed in TBR, confirming the inappropriateness of direct comparison of exercise-related factors regarding the two breeds. This study identified a number of risk factors for MSI associated with training. Risk factors related to the animal and training methods do not seem to account for significant variations in MSI IR. Racing intensity and race numbers were shown to be a protective factor for MSI. Recent medical history could be used to identify horses at increased risk. Our results could provide initial data for monitoring the effect of future strategies and interventions aimed at reducing STBR wastage.

## Abbreviations

MSI: Musculoskeletal injuries; STBR: Standardbred racehorses; TBR: Thoroughbred racehorses; CI: Interval of confidence; IR: Incidence rate; IRR: Incidence rate ratio; PBFx: Pedal bone fracture; DIPA: Distal interphalangeal arthropathy; PPFx: Proximal phalanx fracture; FA: Traumatic fetlock arthropathy; DSI: Digital sheath injury; MTFx: Proximal metacarpal/metatarsal fractures; SLI: Suspensory ligament injury; ASBFx: Apical sesamoid bones fracture; SDFT: Superficial digital flexors tendonitis; CJL: Carpal joint lameness; TL: Tarsal lameness; TsFx: Tibia stress fractures; MFTJS: Medial femorotibial joint synovitis; SCRIL: Sacroiliac joint lameness; PsFx: Pelvic stress fracture.

## Competing interests

The authors declare that they have no competing interest.

## Authors’ contributions

AB conceived the study, and participated in its design and coordination and drafted the manuscript. MB participated in the design of the study and contributed to drafting the manuscript. MG conceived the statistical method to analyse the dataset. MG and FR performed the analysis and drafted the section regarding the statistical analysis. FR organized the tables and data. All authors read and approved the final manuscript.

## Supplementary Material

Additional file 1 Description of categories of injuries. Click here for file

Additional file 2: Table S1 Incidence rates in the various categories of MSI. Category, number and proportion of injuries, incidence rate (IR) per 100 horse-months at risk (with 95% confidence intervals) for young (18 months to 4 years old), adult (>4 years old) and overall. P-values for the comparisons of young *vs* adult horses. Click here for file

Additional file 3 Univariable regression for risk factors studied on the whole population. Click here for file

Additional file 4 Multivariable regression for risk factors studied on the whole population. Click here for file

Additional file 5: Table S2 Performance characteristics of drivers considered in our study. Source by ANAGT ippica.biz web site. Click here for file
