# Supplementary material for: Epidemiology of musculoskeletal injuries in a population of harness Standardbred racehorses in training
Source: BMC Vet Res. 2014 Jan 10;10:11. doi: 10.1186/1746-6148-10-11 (PMC3922780; doi:10.1186/1746-6148-10-11)
Supplement: Additional file 1 — Description of categories of injuries. [file 1746-6148-10-11-S1.doc]

Additional file 1: Description of categories of injuries

| Categories of injuries | Median Lameness score | Diagnostic imaging | Diagnostic analgesia | Clinical assessment |
| --- | --- | --- | --- | --- |
| SLI (N = 89) | 3/5 | Ultrasonography assessment: | Positive response to direct infiltration of the suspensory ligament insertion (sub-carpal and sub-tarsal regions) (N = 12) | Swollen limb (N = 75) |
|  |  | Core lesions at the body (N = 30) |  |  |
|  |  | Insertional lesions at the sub-tarsal and sub-carpal region (N = 12) |  | Painful response to suspensory body palpation (N = 34) |
|  |  |  |  | Painful at passive flexion of the fetlock (N = 67) |
| (suspensory ligament injury) |  | Suspensory branch lesions (N = 47) |  |  |
|  |  | X-ray assessment: |  |  |
|  |  |  |  | Painful response to insertional thumb pressure test (N = 12) |
|  |  | Sclerosis at the proximal palmar aspect of III MTC bone (N = 8) |  |  |
|  |  |  |  | Painful response to suspensory branch palpation (N = 45) |
|  |  | Avulsion fractures at the proximal palmar cortex of III MTC bone (N = 3) |  |  |
|  |  |  |  | Brakes to gallop in straight during fast training (N = 10) |
| FA (N = 43) | 2/5 | X-ray assessment: | Positive response to intra-synovial analgesia | Joint distension (N = 27) |
|  |  | None (N = 4) |  |  |
| (traumatic fetlock arthropathy) |  | Flattening of the palmar/plantar condyle on the lateral view (N = 36) | (N = 26) | Positive response to flexion test (N = 43) |
|  |  |  | Positive response to low-4-points-nerves-block and negative to intra-synovial analgesia (N = 17) |  |
|  |  | Subchondral bone sclerosis on lateral view (N = 37) |  |  |
|  |  |  |  | Drifting away during training (N = 27) |
|  |  | Reduced joint space on the frontal view (N = 9) |  |  |
|  |  | Fragmentation of the dorsal border of P1 (N = 6) |  |  |
| DSI (N = 21) | 4/5 | Ultrasonography assessment: | None | Direct impaction with the foot to the palmar aspect of the fetlock during fast training (N = 22) |
| (digital sheath injury) |  |  |  |  |
|  |  | Concurrent traumatic lesion of the superficial digital flexor tendon (N = 14) |  |  |
|  |  |  |  | (20 cases resulted in open laceration of the digital sheath) |
| CJL (N = 30) | 2/5 | X-ray assessment: | Positive response to intra-synovial analgesia of the middle carpal joint (N = 30) | Bilateral forelimb plaiting (N = 9) |
| (carpal joint lameness) |  | None (N = 3) |  |  |
|  |  | Third carpal bone sclerosis (N = 24) |  | Drifting-away during training in straight (N = 21) |
|  |  | Focal osteolysis in the dorsal cortex of the third carpal bone (N = 11) |  |  |
|  |  |  |  | Middle carpal joint effusion (N = 24) |
|  |  | Slab fracture of the third carpal bone (N = 5) |  |  |
|  |  | Fragmentation of the radial, intermediate and third carpal bone (N = 4) |  | Positive flexion test (N = 30) |
| SDFT (N = 76) | 1/5 | Ultrasonography assessment: | None | Bowed tendon (N = 67) |
|  |  |  |  | Swollen pastern (N = 9) |
|  |  | Core lesions at the mid-metacarpal area (N = 11) |  | Painful response at palpation (N = 75) |
| (superficial digital flexor tendonitis) |  | Lesions at the medial or lateral margin of the tendon cross-sectional area in the metacarpal area (N = 52) |  |  |
|  |  |  |  | Positive flexion test (N = 8) |
|  |  | Lesions in the metatarsal area (N = 4) |  |  |
|  |  |  |  | (only done for lesions at the pastern) |
|  |  | Lesions at the distal branches of superficial digital flexor tendon at the pastern (N = 9) |  |  |
| MFTJS (N = 15) | 2/5 | Ultrasonography assessment: | Positive response to intra-synovial medial femorotibial joint analgesia (N = 8) | Brakes to gallop at fast training in curves (N = 9) |
|  |  |  |  | Effusion of the medial femorotibial joint (N = 15) |
|  |  | Synovial proliferation and effusion at the medial recess of the femorotibial joint |  |  |
|  |  | (N = 12) |  |  |
| (medial femorotibial joint synovitis) |  |  |  |  |
|  |  | Meniscal damage/protrusions (N = 6) |  |  |
|  |  |  |  | Painful response at palpation of patellar ligaments (N = 2) |
|  |  | Lesions to the patellar ligaments (N = 3) | (not done in all cases) |  |
| PPFx (N = 26) | 3/5 | X-ray assessment: | None | Positive flexion test (N = 18) |
|  |  | Short sagittal fractures (N = 14) |  |  |
|  |  | Frontal slab fractures (N = 7) |  | (only done in cases of short sagittal fracture when lameness score was 1/5 to 2/5) |
| (proximal phalanx fracture) |  | Sagittal and oblique complete fractures (N = 5) |  |  |
| PBFx (N = 12) | 3/5 | X-ray assessment: | Positive response to the abaxial sesamoid nerve block (N = 12) | Positive response with hoof tester (N = 11) |
|  |  | Type II fractures at the lateral palmar process (N = 8) |  |  |
| (pedal bone fracture) |  |  |  |  |
|  |  | Type II fractures at the medial palmar process (N = 4) |  |  |
| DIPA (N = 8) | 1/5 | X-ray assessment: | Positive response to intra-synovial analgesia (N = 8) | Brakes to gallop during fast- training in straight (N = 6) |
| (distal interphalangeal joint arthropathy) |  |  |  |  |
|  |  | None (N = 8) |  |  |
|  |  |  |  | Drifting away during training in straight (N = 6) |
| ASBFx (N = 29) | 4/5 | X-ray assessment: | None | Sudden lameness at the end of race or after fast training (N = 28) |
|  |  | Lateral sesamoid bone fractures (N = 10) |  |  |
| (apical sesamoid bone fracture) |  | Medial sesamoid bone fractures (N = 19) |  |  |
|  |  | Ultrasonography assessment: |  |  |
|  |  |  |  | Painful response at palpation of the abaxial surface of the affected sesamoid bone (N = 25) |
|  |  | Distraction of the sesamoid apex at the abaxial surface of the proximal sesamoid bone (N = 25) |  |  |
| TsFx (N = 9) | 4/5 | X-ray assessment: | None | Sudden lameness at the end of fast-training (N = 9) |
|  |  | Monocortical fractures at the proximo-lateral aspect of the tibia (N = 6) |  |  |
| (tibial stress fracture) |  | Monocortical fractures at the mid-diaphysis of the tibia (N = 3) |  |  |
|  |  | Scintigraphic assessment: |  |  |
|  |  | Increase Radiopharmaceutical Uptake at the mid-diaphysis of the tibia (N = 2) |  |  |
| SCRIL (N = 13) | 1/5 | Ultrasonography assessment: | None | Brakes to gallop during fast- exercise in curve (N = 9) |
|  |  | Ventral sacroiliac joint sub-luxation (N = 4) |  |  |
|  |  | Fractures of the sacral facets (N = 5) |  |  |
| (sacroiliac joint arthropathy) |  | Sacroiliac joint ligaments injuries (N = 4) |  | Drifting away during training in straight (N = 11) |
|  |  |  |  | Painful response at deep palpation of the sacral tuberosities (N = 13) |
| PsFx (N = 14) | 3/5 | Ultrasonography assessment: | None | Sudden lameness during fast training (N = 14) |
|  |  | Ileum wing fractures (N = 10) |  |  |
|  |  | Ischiatic fractures (N = 4) |  |  |
| (pelvic stress fracture) |  |  |  |  |
| Curb (N = 11) | 3/5 | Ultrasonography assessment: | None | Sudden lameness after fast- training (N = 9) |
|  |  | Peritendinous/periligamentous tissue injury in the plantar aspect of the hock (N = 11) |  |  |
|  |  |  |  | Swollen profile at the proximal plantar aspect of the hock (N = 11) |
| TL (N = 20) | 2/5 | X-ray assessment: | Positive response to intra-synovial tarso-metatarsal joint anaesthesia (N = 17) | Brakes to gallop during fast- training in curve (N = 6) |
|  |  | Tarsometatarsal joint artropathy (N = 9) |  |  |
| (tarsal lameness) |  | Intertarsal joint artropathy (N = 13) |  | Drifting away during training (N = 15) |
|  |  | Talocalcaneal joint artropathy (N = 3) |  |  |
|  |  | Third tarsal bone slab fracture (N = 3) |  |  |
|  |  |  | not done in all cases | Positive flexion test (N = 18) |
|  |  | Third tarsal bone sagittal fracture (N = 1) |  |  |
| MTFx (N = 14) | 3/5 | X-ray assessment: | None | Sudden lameness after fast training (N = 14) |
| (third metacarpal/ |  | Incomplete fracture of the proximal aspect of third metacarpal bone (N = 3) |  |  |
|  |  |  |  | Painful reaction at palpation of the proximal metacarpal or metatarsal bone (N = 11) |
| metatarsal fracture) |  |  |  |  |
|  |  | Incomplete fracture of the proximal aspect of third metatarsal bone (N = 11) |  |  |

Clinical findings defining the different categories of exercise-related MSI in a cohort of harness STBR in training, observed over 4 years.
