# Supplementary material for: Epidemiology of musculoskeletal injuries in a population of harness Standardbred racehorses in training
Source: BMC Vet Res. 2014 Jan 10;10:11. doi: 10.1186/1746-6148-10-11 (PMC3922780; doi:10.1186/1746-6148-10-11)
Supplement: Additional file 2: Table S1 — Incidence rates in the various categories of MSI. Category, number and proportion of injuries, incidence rate (IR) per 100 horse-months at risk (with 95% confidence intervals) for young (18 months to 4 years old), adult (>4 years old) and overall. P-values for the comparisons of young vs adult horses. [file 1746-6148-10-11-S2.docx]

| **Categories of injuries** | **Cases (%) n=429** | **Months at risk (%)**  **n= 8961** | **Cases** | | **Months at risk** | | **IR –YOUNG**  **( 95% CI)** | **IR-ADULT**  **(95%CI)** | **p-value** | **Overall IR**  **(95%CI)** |
| --- | --- | --- | --- | --- | --- | --- | --- | --- | --- | --- |
|  |  |  | **YOUNG**  **(%)** | **ADULT**  **(%)** | YOUNG  (%) | ADULT  (%) |  |  |  |  |
| **SLI** | 89 (20.7) | 2296 (25.6) | 64 (71.9) | 25 (28.1) | 1583 (68.9) | 713 (31.1) | 0.919  (0.720-1.174) | 1.251  (0.845-1.851) | 0.192 | 0.993  (0.798-1.222) |
| **SDFT** | 76 (17.7) | 1861 (20.8) | 59 (77.6) | 17 (22.4) | 1440 (77.4) | 421(22.6) | 0.847  (0.657-1.094) | 0.850  (0.529-1.368) | 0.990 | 0.848  (0.668-1.062) |
| **FA** | 43 (10.0) | 1274 (14.2) | 33 (76.7) | 10 (23.3) | 941 (73.9) | 333 (26.1) | 0.474  (0.337-0.667) | 0.500  (0.269-0.930) | 0.881 | 0.480  (0.347-0.646) |
| **CJL** | 30 (7.0) | 647 (7.2) | 29 (96.7) | 1 (3.3) | 640 (98.9) | 7 (1.1) | 0.417  (0.289-0.599) | 0.050  (0.007-0.355) | 0.037 | 0.335  (0.226-0.478) |
| **ASBFx** | 29 (6.8) | 667 (7.4) | 24 (82.8) | 5 (17.2) | 593 (88.9) | 74 (11.1) | 0.345  (0.231-0.514) | 0.250  (0.104-0.601) | 0.514 | 0.324  (0.217-0.465) |
| **PPFx** | 26 (5.8) | 562 (6.3) | 19 (76) | 6 (24) | 442 (78.6) | 120 (21.4) | 0.273  (0.174-0.428) | 0.300  (0.135-0.668) | 0.839 | 0.279  (0.181-0.412) |
| **DSI** | 21 (4.9) | 488 (5.4) | 16 (76.2) | 5 (23.8) | 401 (82.2) | 87 (17.8) | 0.230  (0.141-0.375) | 0.250  (0.104-0.601) | 0.869 | 0.234  (0.145-0.358) |
| **TL** | 20 (4.7) | 458 (5.1) | 17 (85) | 3 (15) | 414 (90.4) | 44 (9.6) | 0.244  (0.152-0.393) | 0.150  (0.048-0.465) | 0.437 | 0.223  (0.136-0.345) |
| **MFTJS** | 15 (3.5) | 369 (4.1) | 14 (93.3) | 1 (6.7) | 339 (91.9) | 30 (8,1) | 0.201  (0.119-0.340) | 0.050  (0.007-0.355) | 0.990 | 0.167  (0.094-0.276) |
| **PsFX** | 14 (3.3) | 280 (3.1) | 11 (78.6) | 3 (21.4) | 233 (83.2) | 47 (16.8) | 0.158  (0.088-0.285) | 0.150  (0.048-0.465) | 0.937 | 0.156  (0.085-0.262) |
| **MTFX** | 14 (3.3) | 321 (3.6) | 12 (85.7) | 2 (14.3) | 308 (96) | 13 (4) | 0.172  (0.098-0.304) | 0.100  (0.025-0.400) | 0.476 | 0.156  (0.085-0.262) |
| **SCRIL** | 13 (3.0) | 407 (4.5) | 8 (61.5) | 5 (38.5) | 233 (57.2) | 174 (42.8) | 0.115  (0.057-0.230) | 0.250  (0.104-0.601) | 0.172 | 0.145  (0.077-0.248) |
| **PBFX** | 12 (2.8) | 312 (3.5) | 10 (83.3) | 2 (17.7) | 268 (85.9) | 44 (14.1) | 0.144  (0.077-0.267) | 0.100  (0.025-0.400) | 0.641 | 0.134  (0.069-0.234) |
| **Curb** | 11 (2.6) | 275 (3.1) | 11 (100) | 0 (0) | 275 (100) | 0 (0) | 0.158  (0.088-0.285) | 0  Not available | 0.991 | 0.123  (0.061-0.220) |
| **TsFX** | 9 (2.1) | 168 (1.9) | 8 (88.9) | 1 (11.1) | 125 (74.4) | 43 (25.6) | 0.115  (0.057-0.230) | 0.050  (0.007-0.355) | 0.433 | 0.100  (0.046-0.191) |
| **DIPA** | 8 (1.9) | 232 (2.6) | 6 (75) | 2 (25) | 168 (72.4) | 74 (27.6) | 0.086  (0.039-0.192) | 0.100  (0.025-0.400) | 0.855 | 0.089  (0.039-0.176) |
| **Total** | 429 | 8961 | 341 (79.5) | 88 (20.5) |  |  |  |  |  | 4.787 |
