# Supplementary material for: Epidemiology of musculoskeletal injuries in a population of harness Standardbred racehorses in training
Source: BMC Vet Res. 2014 Jan 10;10:11. doi: 10.1186/1746-6148-10-11 (PMC3922780; doi:10.1186/1746-6148-10-11)
Supplement: Additional file 3 — Univariable regression for risk factors studied on the whole population. [file 1746-6148-10-11-S3.doc]

**Additional file3:** Univariable regression for risk factors studied on the whole population

| Variables | Category | MSI | Months at risk (%) | IR | IR 95% CI | IRR | IRR 95% CI | p-value |
| --- | --- | --- | --- | --- | --- | --- | --- | --- |
|  |  | Cases (%) |  |  |  |  |  |  |
|  |  | n = 429 | n = 8961 |  |  |  |  |  |
| AGE | YOUNG | 341 (79.5) | 6962 (77.7) | 4.90 | 4.40 - 5.45 | 1.00 | - | REF |
|  | ADULT | 88 (20.5) | 1999 (22.3) | 4.40 | 3.57 - 5.43 | 0.90 | 0.71 - 1.14 | 0.372 |
| GENDER | MALE | 181 (42.2) | 3680 (41.1) | 4.92 | 4.25 - 5.69 | 1.00 | - | REF |
|  | FEMALE | 181 (42.2) | 3371 (37.6) | 5.37 | 4.64 - 6.21 | 1.09 | 0.89 - 1.34 | 0.404 |
|  | GELDING | 67 (15.6) | 1910 (21.3) | 3.51 | 2.76 - 4.46 | 0.71 | 0.54 - 0.94 | 0.018 |
| DRIVER | DRIVER 1 | 23 (5.4) | 393 (4.4) | 5.85 | 3.89 - 8.81 | 1.00 | - | REF |
|  | DRIVER 2 | 94 (21.9) | 1570 (17.5) | 5.99 | 4.89 - 7.33 | 1.02 | 0.65 - 1.61 | 0.922 |
|  | DRIVER 3 | 34 (7.9) | 1014 (11.3) | 3.35 | 2.40 - 4.69 | 0.57 | 0.34 - 0.97 | 0.039 |
|  | DRIVER 4 | 69 (16.1) | 1603 (17.9) | 4.30 | 3.40 - 5.45 | 0.74 | 0.46 - 1.18 | 0.202 |
|  | DRIVER 5 | 15 (3.5) | 351 (3.9) | 4.27 | 2.58 - 7.09 | 0.73 | 0.38 - 1.40 | 0.343 |
|  | DRIVER 6 | 33 (7.7) | 772 (8.6) | 4.27 | 3.04 - 6.01 | 0.73 | 0.43 - 1.24 | 0.247 |
|  | DRIVER 7 | 48 (11.2) | 813 (9.1) | 5.90 | 4.45 - 7.83 | 1.01 | 0.61 - 1.66 | 0.972 |
|  | DRIVER 8 | 81 (18.9) | 1817 (20.3) | 4.46 | 3.59 - 5.54 | 0.76 | 0.48 - 1.21 | 0.249 |
|  | DRIVER 9 | 12 (2.8) | 309 (3.4) | 3.88 | 2.21 - 6.84 | 0.66 | 0.33 - 1.33 | 0.249 |
|  | DRIVER 10 | 20 (4.7) | 319 (3.6) | 6.27 | 4.04 - 9.72 | 1.07 | 0.59 - 1.95 | 0.822 |
| RACING SPEED | Not qualified STBR | 74 (17.2) | 719 (8.0) | 10.29 | 8.20 - 12.93 | 1.00 | - | REF |
|  | Low level STBR | 58 (13.5) | 912 (10.2) | 6.36 | 4.92 - 8.23 | 0.62 | 0.44 - 0.87 | 0.006 |
|  | Medium level STBR | 170 (39.6) | 3888 (43.4) | 4.37 | 3.76 - 5.08 | 0.42 | 0.32 - 0.56 | <0.001 |
|  | Elite STBR | 106 (24.7) | 2902 (32.4) | 3.65 | 3.02 - 4.42 | 0.35 | 0.26 - 0.48 | <0.001 |
|  | Top STBR | 21 (4.9) | 540 (6.0) | 3.89 | 2.54 - 5.96 | 0.38 | 0.23 - 0.61 | <0.001 |
| RACING INTENSITY | 0-4 races/year | 176 (41) | 1818 (20.3) | 9.68 | 8.35 - 11.22 | 1.00 | - | REF |
|  | 5-11 races/year | 127 (29.6) | 2810 (31.4) | 4.52 | 3.80 - 5.38 | 0.47 | 0.37 - 0.59 | <0.001 |
|  | 12-22 races/year | 118 (27.5) | 3886 (43.4) | 3.04 | 2.54 - 3.64 | 0.31 | 0.25 - 0.40 | <0.001 |
|  | 23-32 races/year | 8 (1.9) | 447 (5.0) | 1.79 | 0.90 - 3.58 | 0.18 | 0.09 - 0.38 | <0.001 |
| Variables studied on subsets of the population | | | | | | | | |
| RACING SHOD | Shod | 251 (65) | 5544 (64.6) | 4.53 | 4.00 - 5.12 | 1.00 | - | REF |
| (subset of horses which ran at least one race) |  |  |  |  |  |  |  |  |
|  | Unshod | 135 (35) | 3036 (35.4) | 4.45 | 3.76 - 5.26 | 0.98 | 0.80 - 1.21 | 0.866 |
| cases = 386 |  |  |  |  |  |  |  |  |
| months at risk = 8580 |  |  |  |  |  |  |  |  |
| MEDICAL TREATMENTS | No | 260 (60.6) | 6543 (80.1) | 3.97 | 3.52 - 4.49 | 1.00 | - | REF |
| 30d prior to INJ |  |  |  |  |  |  |  |  |
| (subset of horses with at least one injury) |  |  |  |  |  |  |  |  |
| cases =429 |  |  |  |  |  |  |  |  |
| months at risk =8164 |  |  |  |  |  |  |  |  |
|  | Yes | 169 (39.4) | 1621 (19.9) | 10.43 | 8.97 -12.12 | 2.63 | 2.16-3.18 | <0.001 |

Results of univariable Poisson regression investigating risk factors for MSI in STBR, with incidence rate, 95% confidence interval of IR, incidence rate ratio (IRR), 95% confidence interval of IRR and p-values. Data drawn from our entire population, including 429 cases of MSI, over 8961 months at risk.
