# Supplementary material for: Epidemiology of musculoskeletal injuries in a population of harness Standardbred racehorses in training
Source: BMC Vet Res. 2014 Jan 10;10:11. doi: 10.1186/1746-6148-10-11 (PMC3922780; doi:10.1186/1746-6148-10-11)
Supplement: Additional file 4 — Multivariable regression for risk factors studied on the whole population. [file 1746-6148-10-11-S4.doc]

Additional file 4: Multivariable regression for risk factors studied on the whole population

| Variable | Category | IRR | IRR 95% CI | p-value |
| --- | --- | --- | --- | --- |
| RACING SPEED | Not qualified STBR | 1.00 | - | REF |
|  | Low level STBR | 1.04 | 0.72 - 1.51 | 0.828 |
|  | Medium level STBR | 0.91 | 0.66 - 1.25 | 0.550 |
|  | Elite STBR | 0.81 | 0.57 - 1.15 | 0.236 |
|  | Top STBR | 0.81 | 0.48 - 1.36 | 0.416 |
| GENDER | MALE | 1.00 | - | REF |
|  | FEMALE | 1.08 | 0.87 - 1.33 | 0.487 |
|  | GELDING | 0.85 | 0.64 - 1.13 | 0.261 |
| RACING INTENSITY | 0 to 4 races a year | 1.00 | - | REF |
|  | 5 to 11 races a year | 0.50 | 0.39 - 0.65 | <0.001 |
|  | 12 to 22 races a year | 0.35 | 0.27 - 0.46 | <0.001 |
|  | 23 to 32 races a year | 0.21 | 0.10 – 0.43 | <0.001 |

Results of multivariable mixed Poisson regression, with DRIVER as random effect, with Incidence Rate Ratio (IRR), 95% confidence interval of IRR, and p-values. Data drawn from our entire population, including 429 cases of MSI, over 8961 months at risk.
