# Supplementary material for: Epidemiology of musculoskeletal injuries in a population of harness Standardbred racehorses in training
Source: BMC Vet Res. 2014 Jan 10;10:11. doi: 10.1186/1746-6148-10-11 (PMC3922780; doi:10.1186/1746-6148-10-11)
Supplement: Additional file 5: Table S2 — Performance characteristics of drivers considered in our study. Source by ANAGT ippica.biz web site. [file 1746-6148-10-11-S5.docx]

|  | **2008** | | | **2009** | | | **2010** | | | **2011** | | |
| --- | --- | --- | --- | --- | --- | --- | --- | --- | --- | --- | --- | --- |
|  | *Races* | *% first place* | *Earning (€)* | *Races* | *% first place* | *Earning (€)* | *Races* | *% first place* | *Earning (€)* | *Races* | *% first place* | *Earning (€)* |
| ***Driver 1*** | 138 | 8,76 | 83.288 | 137 | 8,76 | 83.288 | 154 | 9,09 | 86.704 | 156 | 12,18 | 133.146 |
| ***Driver 2*** | 154 | 8,44 | 75.902 | 156 | 8,33 | 89.463 | 168 | 11,31 | 89.463 | 208 | 10,58 | 124.890 |
| ***Driver 3*** | 192 | 3,13 | 64.639 | 168 | 2,38 | 50.516 | 130 | 2,31 | 35.008 | 66 | 1,52 | 5.530 |
| ***Driver 4*** | 821 | 27,53 | 2.027.194 | 611 | 21,60 | 1.129.306 | 715 | 22,94 | 1.015.593 | 583 | 20,06 | 1.085.780 |
| ***Driver 5*** | 191 | 5,24 | 80.508 | 19 | 5,26 | 12.394 | 34 | 5,88 | 16.898 | 43 | 13,95 | 28.460 |
| ***Driver 6*** | 186 | 5,38 | 69.497 | 106 | 9,43 | 55.278 | 257 | 3,11 | 93.986 | 45 | 0 | 10.580 |
| ***Driver 7*** | 279 | 7,17 | 129.486 | 265 | 8,30 | 224.586 | 341 | 6,16 | 198.889 | 267 | 6,37 | 181.572 |
| ***Driver 8*** | 776 | 20,88 | 1.338.932 | 610 | 20,33 | 1.311.683 | 506 | 16,80 | 945.331 | 466 | 17,81 | 731.964 |
| ***Driver 9*** | 173 | 1,16 | 34.332 | 182 | 0 | 34.592 | 155 | 0,65 | 25.662 | 120 | 1,67 | 29.254 |
| ***Driver 10*** | 14 | 0 | 3.194 | 59 | 3,39 | 14.818 | 86 | 5,81 | 25.816 | 107 | 2,8 | 25.070 |
